# Fully automated brain resection cavity delineation for radiation target volume definition in glioblastoma patients using deep learning

**DOI:** 10.1186/s13014-020-01553-z

**Published:** 2020-05-06

**Authors:** Ekin Ermiş, Alain Jungo, Robert Poel, Marcela Blatti-Moreno, Raphael Meier, Urspeter Knecht, Daniel M. Aebersold, Michael K. Fix, Peter Manser, Mauricio Reyes, Evelyn Herrmann

**Affiliations:** 1 Department of Radiation Oncology, Inselspital, Bern University Hospital, and University of Bern, Freiburgstrasse, 3010 Bern, Switzerland; 2 Insel Data Science Center, Inselspital, Bern University Hospital, Bern, Switzerland; 3 ARTORG Center for Biomedical Research, University of Bern, Bern, Switzerland; 4 Institute for Diagnostic and Interventional Neuroradiology, Inselspital, Bern University Hospital, and University of Bern, Bern, Switzerland; 5 Division of Medical Radiation Physics and Department of Radiation Oncology, Inselspital, Bern University Hospital, and University of Bern, Bern, Switzerland

**Keywords:** Glioblastoma, Automatic segmentation, Deep learning, Target definition, MRI

## Abstract

**Background:**

Automated brain tumor segmentation methods are computational algorithms that yield tumor delineation from, in this case, multimodal magnetic resonance imaging (MRI). We present an automated segmentation method and its results for resection cavity (RC) in glioblastoma multiforme (GBM) patients using deep learning (DL) technologies.

**Methods:**

Post-operative, T1w with and without contrast, T2w and fluid attenuated inversion recovery MRI studies of 30 GBM patients were included. Three radiation oncologists manually delineated the RC to obtain a reference segmentation. We developed a DL cavity segmentation method, which utilizes all four MRI sequences and the reference segmentation to learn to perform RC delineations. We evaluated the segmentation method in terms of Dice coefficient (DC) and estimated volume measurements.

**Results:**

Median DC of the three radiation oncologist were 0.85 (interquartile range [IQR]: 0.08), 0.84 (IQR: 0.07), and 0.86 (IQR: 0.07). The results of the automatic segmentation compared to the three different raters were 0.83 (IQR: 0.14), 0.81 (IQR: 0.12), and 0.81 (IQR: 0.13) which was significantly lower compared to the DC among raters (chi-square = 11.63, *p* = 0.04). We did not detect a statistically significant difference of the measured RC volumes for the different raters and the automated method (Kruskal-Wallis test: chi-square = 1.46, *p* = 0.69). The main sources of error were due to signal inhomogeneity and similar intensity patterns between cavity and brain tissues.

**Conclusions:**

The proposed DL approach yields promising results for automated RC segmentation in this proof of concept study. Compared to human experts, the DC are still subpar.

## Background

Glioblastoma multiforme (GBM) is the most common primary malignant brain tumor. The invasive nature of the disease makes the treatment very challenging which is expressed in the poor prognosis with a 5 year survival rate of 5% [1]. Since the 1980s, it is established that post-operative radiation therapy (RT) improves survival in patients with GBM [2] and RT is part of the multidisciplinary treatment ever since. With an incidence ranging from 2.50 to 5.02 cases per 100.000 person years GBM is one of the major indications for radiation therapy [3–5].

One of the most tedious and time-consuming tasks in radiotherapy planning is target and organ at risk (OAR) contouring. This is still done manually in a slice by slice fashion, using multiple magnetic resonance imaging (MRI) sequences [2]. Besides, manual contouring is associated with a wide variability and low uniformity among different users, here called raters. According to Bondiau et al. [6], the mean time for the analysis and manual delineation of brain structures on a typical MRI study is 86 min. Due to human error and observer bias there are substantial intra- and inter-rater variabilities for both target and OAR definition [2, 7]. For GBM in particular, post-op target definition shows substantial inter-rater variability even amongst advanced experts [8]. In this regard, automated contouring methods would be very useful for RT target volume definition. Fully automatic segmentation, where no interaction of the user is required, has the potential to substantially limit the time for target volume and OAR definition. Additionally, it can introduce a more consistent and reproducible standard for volume definition leading to a better agreement among institutes and possibilities for global implementation.

Auto-segmentation of medical imaging has been a hot topic over the last years [9]. The increased interest is driven by the rise of radiomics, where quantitative assessment on medical imaging requires segmented structures of interest. To objectify the comparison among different auto-segmentation methods, the Brain Tumor Image Segmentation Benchmark (BRATS) challenge was introduced in 2012 [9], which enables researchers to test their auto-segmentation methods on a multi-institutional MRI database of glioma tumors. Since the introduction of BRATS, machine learning (ML) based methods have shown very promising results [10]. A recent trend of deep learning (DL) using convolutional neural networks (CNN) led to the current state of the art auto-segmentation methods able to segment glioma volumes with a Dice Coefficient (DC) reaching 0.9 with respect to “ground truth” [11–13]. Despite the impressive results, multi modality DL-based segmentation methods have not been implemented for automated volume definition in RT.

For a proper implementation of auto-segmentation in RT, besides adequate target definition, OARs also need to be defined. Currently available atlas based segmentations are precarious and especially small structures like the chiasm and optic nerves are challenging to segment [7, 14]. In addition, surgical procedures or the presence of space-occupying lesions cause anatomical deviation, and can affect the quality of automated OAR segmentations [7, 15]. However, several other groups as well as our own, presented that in relation to atlas-based approaches, ML and DL based methods showed improved results of auto-segmentation of anatomical brain structures or subcortical brain structures [15–18].

As for the target definition in GBM, there are currently two important guidelines; one from the European Organization for Research and Treatment of Cancer (EORTC) and the other from the Radiation Therapy Oncology Group (RTOG) [19–22]. Both the EORTC and the RTOG define the gross tumor volume (GTV) as the resection cavity (RC) in addition to the residual enhancing tumor. In the RTOG guideline, surrounding edema should also be included. The current auto-segmentation results for glioma segmentation are mainly based on pre-operative imaging. Since the majority of patients receives surgery prior to RT, the GTV is defined on post-operative imaging and therefore segmentation is more challenging. This applies to auto-segmentation as well as manual segmentation. Not many attempts have been made on auto-segmentation of post-operative MR images. Zeng et al. evaluated the segmentation in pre- and post-operative MR images [23]. Their result showed a median dice coefficient of 0.75 with respect to the reference segmentation. In our previous work published by Meier et al., no significant difference between the postoperative automated segmentation of the residual tumor volume and the reference segmentation was found [24]. Unfortunately, both studies lacked the segmentation of the RC, which is critical in GBM patients as well as in patients with other brain tumors who receive adjuvant RT after resection.

Efficacy in auto-contouring has been shown for OARs and most post-operative target structures [11–18, 23, 24]. The current missing link that enables the physician to define the target is the RC. The aim of the present proof of concept study is to evaluate whether our DL automated segmentation method for RCs in GBM patients is comparable to manual segmentation by experts in the process of RT target volume definition. We assessed the agreement between automated and expert-generated RC segmentations using standard overlap and volumetric metrics. To do so, we developed a DL based auto-segmentation tool for brain tumor RT planning.

## Methods

### Patients

Patients with newly diagnosed and histologically confirmed GBM, who were pre-operatively admitted to our institution between 2012 and 2015, were eligible for this study. Furthermore, patients should have undergone primary tumor resection without previous brain surgery and a complete post-operative MR data set should be available according to our GBM MRI protocol. This includes T1 weighted images with and without contrast, T2 weighted images with contrast and a fluid attenuated inversion recovery (FLAIR) sequence. Furthermore the resection cavity should be clearly present on visual inspection. A total of 30 patients were retrospectively included in the study, which is in line with recommendations for the evaluation of segmentation accuracy from Gibson et al. [25]. All patients received adjuvant concomitant chemo-radiotherapy with Temozolomide.

### MR protocol

MR images were acquired on two different 1.5 T MR scanners (Siemens Avanto and Siemens Area, Siemens, Erlangen/Germany). For all patients the same, standardized MR protocol was applied, including four standard MR sequences that constitute the neuro-oncological MR protocol according to the response assessment in neuro-oncology criteria [26]:

- T1- weighted without contrast (T1w), resolution 256 × 256, 1 mm slice thickness, repetition time (TR) = 1580 and echo time (TE) = 2.67.
- T1-weighted gadolinium enhanced (T1w gadolinium), resolution 256 × 256, 1 mm slice thickness, TR = 2070 and TE = 4.57.
- T2 – weighted (T2w), resolution 256 × 256, 1 mm slice thickness, TR = 3200 and TE = 3.81.
- Fluid-attenuated inversion recovery (FLAIR) images, resolution 192 × 256, 3 mm slice thickness, TR = 8000 and TE = 88.

**Fig. 1:**
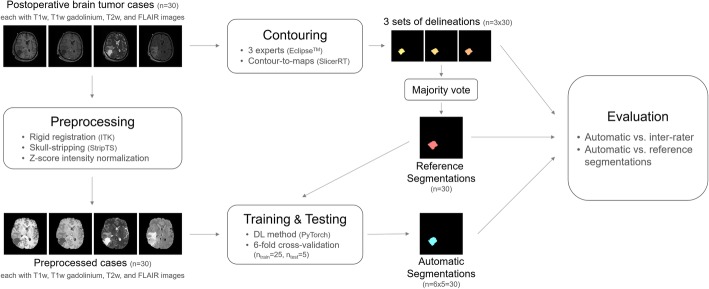
Schematic visualization of the workflow of this study

These sequences were used for the manual and the automatic segmentation process (Fig. 1).

### Manual contouring of the resection cavity

In order to obtain a reference volume to train and evaluate the DL method, the RCs were manually contoured. Manual contouring was performed slice-wise in Eclipse™ version 13.6 (Varian Medical Systems, Inc. Palo Alto, CA, USA) by three independent radiation oncology experts (EH, EE, MB) with with respectively, 9, 3 and 1 years of experience in brain tumor imaging analysis, and familiar with all four MR sequences (T1w, T1w gadolinium, T2w and FLAIR). To improve inter-rater consistency the raters have been instructed by an experienced neuro-radiologist. The RC was defined as the region of liquor isointense signal on T2w MR sequences at the region of the resection, including air pockets determined on T1w and T2w MR sequences as well as the remaining blood collections visible on the T1w MR sequence.

### Data preprocessing

The data set containing the contours was exported as DICOM-RT format. The structure set and the four MRI sequences were then imported into 3D Slicer Version 4.8 (www.slicer.org) with the SlicerRT plugin. Once imported, the polygon structures were translated to label maps, where each voxel was labeled as RC or background.

To prepare the MRI data for DL, multimodal rigid registration was performed. The T1w, T2w and FLAIR MR sequences were rigidly registered to the space of the T1w gadolinium enhanced image [24]. Skull-stripping [27] was performed to eliminate superfluous data, and image intensities were normalized to a standard normal distribution (μ = 0, σ = 1).

To train the DL model, the three expert delineations are fused and referred to as the reference segmentation. The fusion was performed by majority voting, a standard approach where a voxel is considered RC, when 2 or more (out of 3) experts defined the voxel as RC (Fig. 1). The majority voting aims at minimizing confounder effects stemming from any potential expert-specific bias.

### DL architecture

We used a fully-convolutional densely connected architecture which builds on the idea of DenseNet [28] but is adapted for this particular segmentation task. The architecture consists of a contraction-expansion (encoding-decoding) structure with so-called skip-connections introducing shortcuts to additional contraction levels. The contraction-expansion structure aims at capturing the high-level contextual information while the skip-connections enable capturing the local, fine-grained information. The architecture consists of four contraction-expansion levels, each built of one dense block, which itself consists of four densely connected units. Each of these units comprises of batch normalization [29], ReLU activation [30], convolution, and dropout [31]. The transition between contraction or expansion levels is performed by transition-down and transition-up blocks, respectively. Transition-down blocks consist of a dense unit followed by max-pooling. Transition up blocks consist of a bilinear interpolation followed by a convolution and a dense block layer. All convolutions employ 3 × 3 kernels, except the last convolution which has a 1 × 1 kernel. The dropout rate is set to *p* = 0.2. Table 1 lists the channel numbers and spatial resolution after each building block.

**Table 1:** Description of the DL architecture

**Building Block**	**Channels**	**Spatial Resolution**
Input	4	200 × 200
Convolution + Dropout	48	200 × 200
Dense block + Transition down	96	200 × 200
Dense block + Transition down	144	100 × 100
Dense block + Transition down	192	50 × 50
Dense block + Transition down	240	25 × 25
Dense block	288	12 × 12
Transition up + Dense block	336	25 × 25
Transition up + Dense block	288	50 × 50
Transition up + Dense block	240	100 × 200
Transition up + Dense block	192	200 × 200
1 × 1 Convolution	2	200 × 200
Softmax	2	200 × 200

The DL architecture processes the 3D brain volumes as three separate sets of two-dimensional plane-wise orientations, i.e., axial, coronal, sagittal. This results in three 3D predictions of the RC volume based on the axial, coronal and sagittal slices, respectively. The final 3D volume is an average of the three predictions.

Representation of the deep learning architecture in terms of building blocks, channel number, and spatial resolution. The input represents one slice of all four MR images and the output consists of the foreground and background probabilities that define the final segmentation.

### DL training protocol

The proposed DL approach requires a training phase, where the images of the four MR sequences and the manually created reference segmentations are used. Although the cavity is defined in the T1w and T2w images, we use all four sequences to leverage additional information that can be beneficial for training of the DL model. During training, for each MRI sequence we feed batches of 16 slices of random orientation (i.e. axial, coronal, or sagittal). We optimized the cross-entropy loss by the Adam optimizer [32] and used a learning rate of 10^− 4^. The DL training takes approximately 24 h on a NVIDIA Titan Xp graphics processing unit (GPU) with 12 GB memory. The code was implemented in Python 3.6.8 with PyTorch 1.0.1 (pytorch.org).

### Quantitative evaluation and statistics

To evaluate the accuracy of the DL model we adopted a cross-validation scheme, commonly used by supervised learning systems [33]. We performed a six -fold cross validation where the 30 included cases are shuffled randomly and 25 samples are used for training and the remaining five are used for testing, until all 30 cases have an auto-segmented result. We remark that in order to avoid optimizing the model to each cross-evaluation split, we optimized the hyper-parameters on one out of the six splits only.

We compared the automatic segmentations with each of three manual segmentations, as well as the fused reference segmentations (Fig. 1). As a reference of human-level performance, we assessed inter-rater variability. We assessed three different metrics to compare the auto-segmented RC volumes:

1. The absolute volume in cm^3^.
2. The Dice coefficient defined as the volumetric overlap ranging between 0 and 1, where DC = 1 corresponds to perfect agreement.
3. The relative volume error defined as the difference in volume between the auto-segmented RC and the RCs defined by the raters.

For all metrics, we performed a non-parametric Kruskal-Wallis test (α = 0.05) to assess similarity among the non-normal distributions (verified with Shapiro-Wilk test). In case this test showed a significant difference at the group level, the Wilcoxon rank sum tests (unpaired, α = 0.05) was performed for detailed analysis.

## Results

All three raters produced a complete set of contours for all 30 patients. The reference segmentation generated by fusion, were all accepted by the three raters upon review. The average time to contour the RC was 20.7 (± 10.1) minutes. The trained DL models produced automatic segmented RCs for all cases. The DL-based segmentation of one case takes approximately 10 s, and a total of 90 s when including the pre-processing steps on a standard desktop computer.

**Fig. 2:**
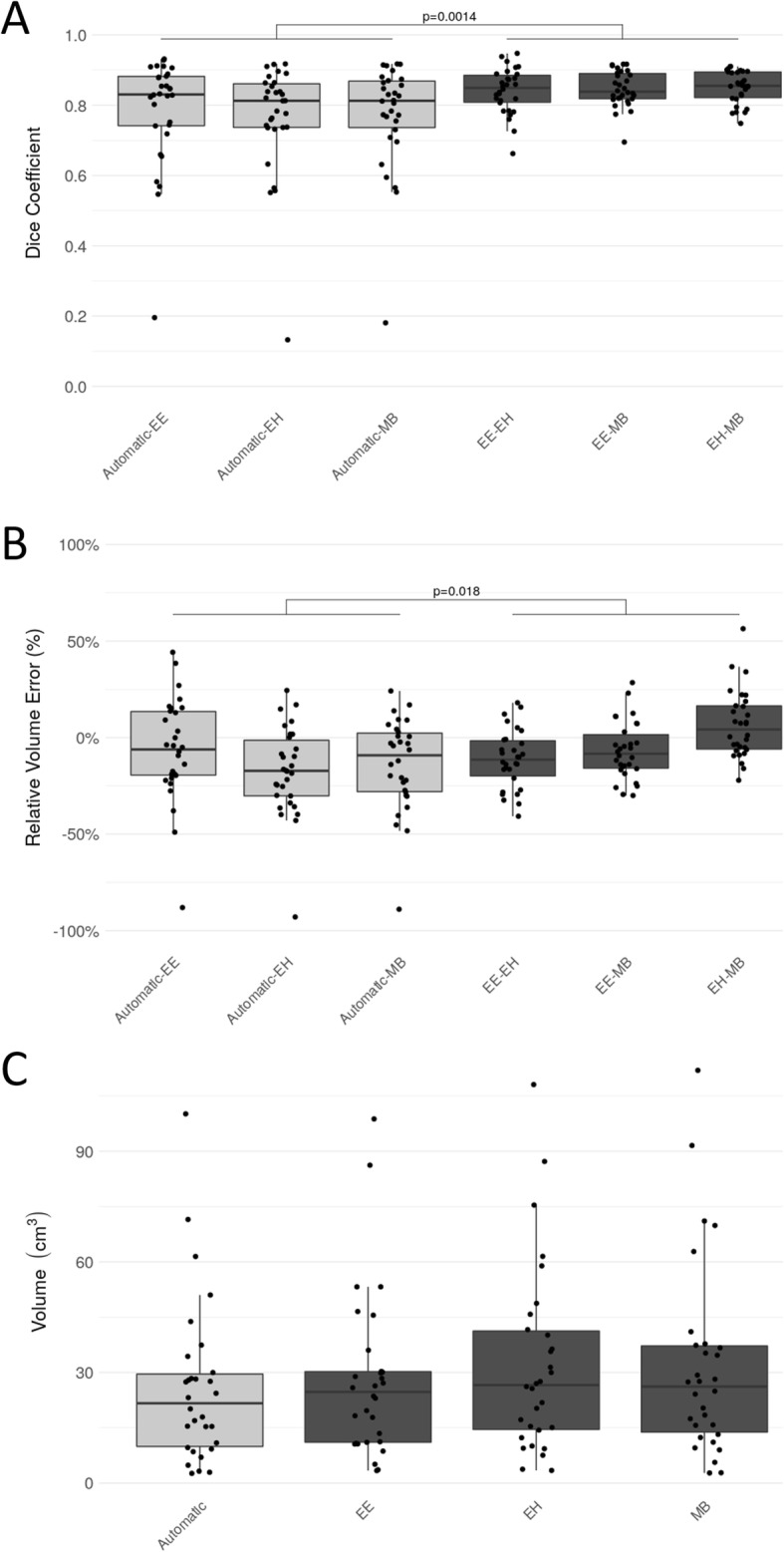
Comparison of the automatic approach and the three experts (EE, EH, MB) in terms of Dice coefficient (**a**), relative volume error (**b**), and absolute volume (**c**) on the cross-evaluated cohort. The light gray boxes on the left represent results of automatic method and the dark gray boxes on the right show the experts. *P*-values indicate the result of the Wilcoxon rank-sum test (α = 0.05) between automatic-rater (Automatic-EE, Automatic-EH, Automatic-MB) and rater-rater (EE-EH, EE-MB, EH-MB) results

**Fig. 3:**
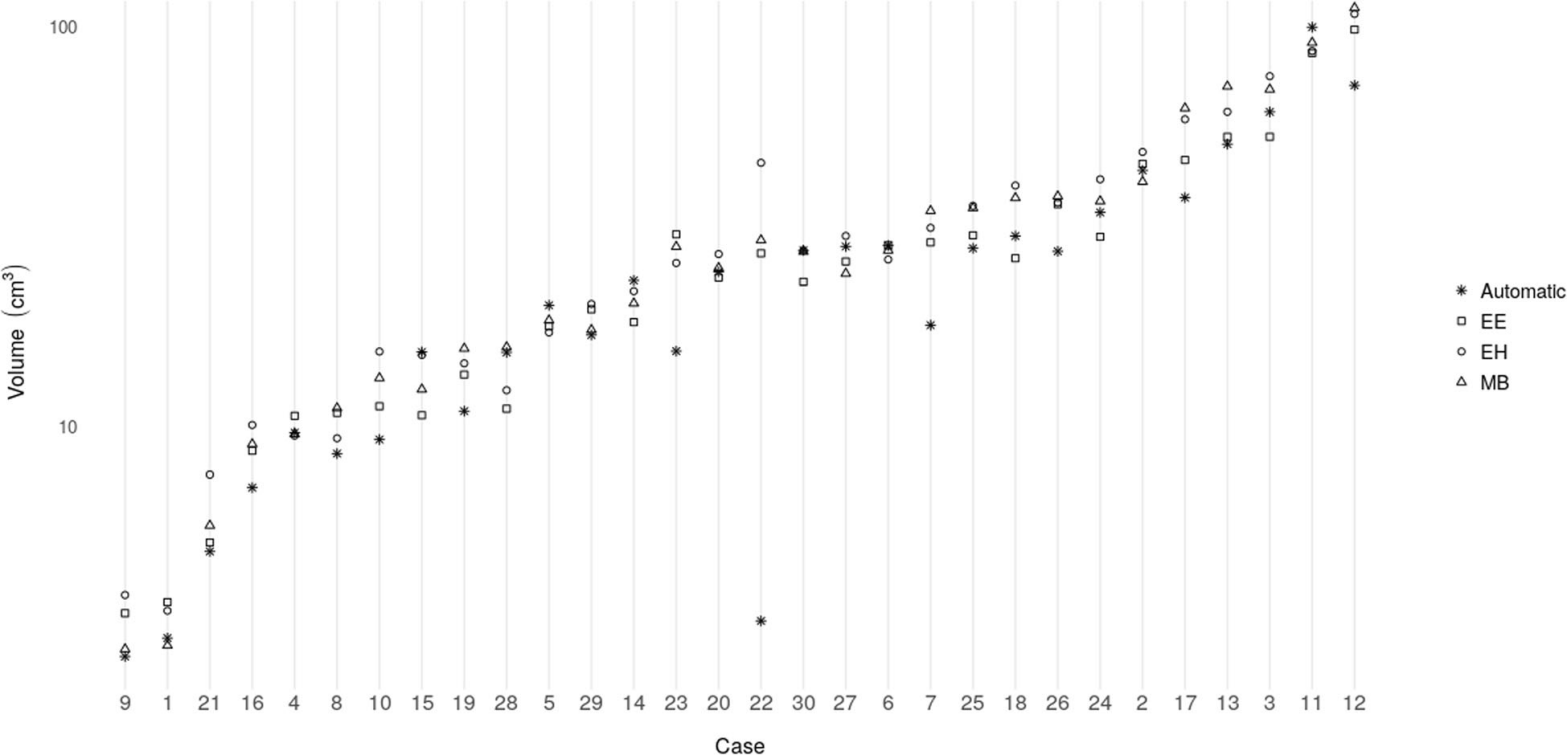
Comparison of the automatic approach and the three experts (EE, EH, MB) in terms of measured resection cavity volume for each case in the dataset. Note the logarithmic scale of the y-axis

**Fig. 4:**
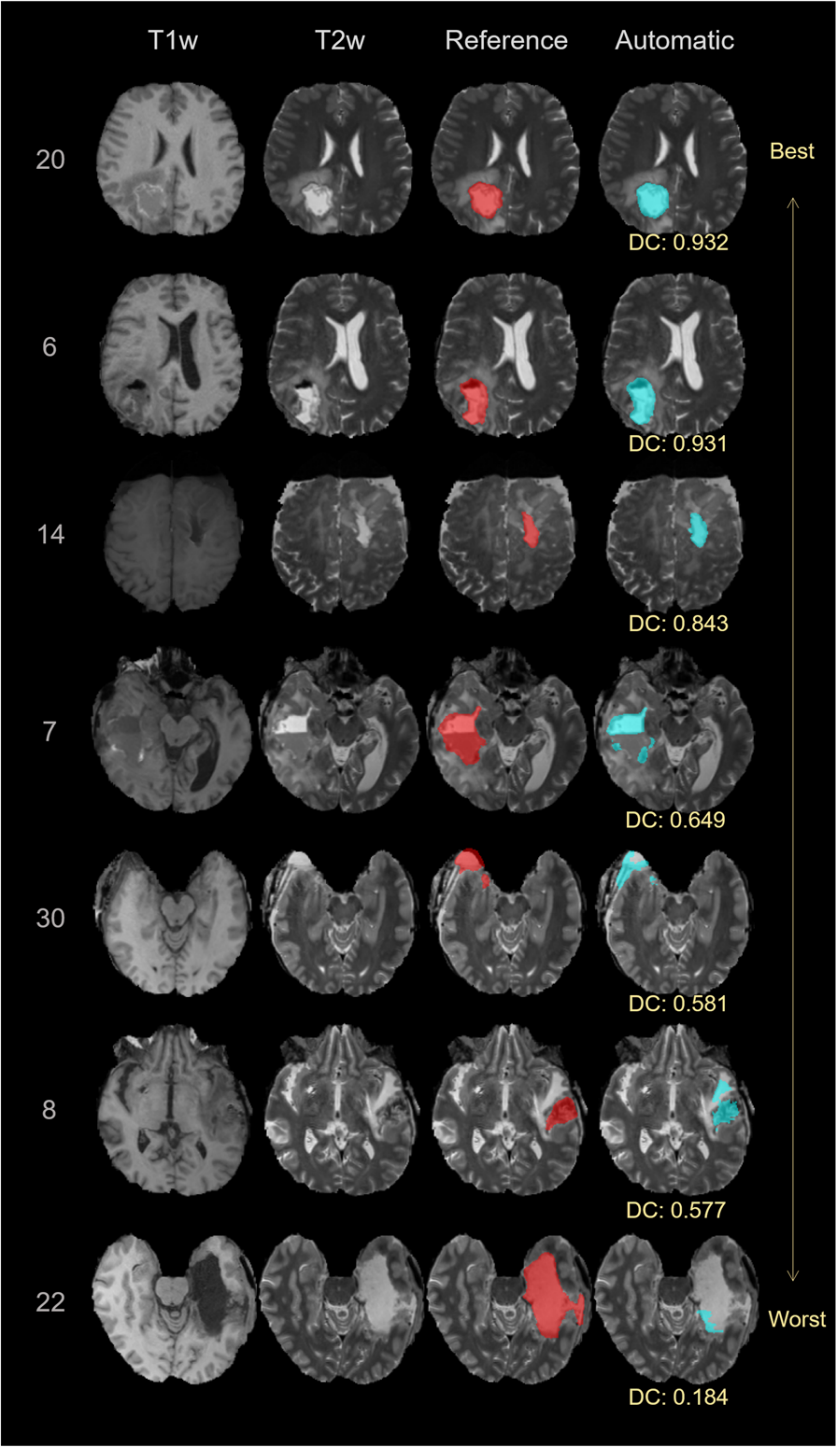
Representative axial slices of the produced segmentations in comparison to the expert consensus. The rows correspond to different cases and are listed according to the segmentation performance in terms of Dice coefficient (DC). The columns show the T1-weighted (T1w) image, the T2-weighted (T2w) image, the expert consensus (reference) and the automatic segmentation (as overlay on the T2w)

The DC and the relative volume error of the different pairings of expert raters and the automatic segmentation are listed in Table 2. The median overall DC among the raters was 0.85 (interquartile range [IQR]:0.07). The median DC between the automatic segmented RCs and the fused reference segmentation was 0.84 (IQR: 0.10), and slightly lower than the agreement among raters. In terms of relative volume error, we found a median error of − 13.17%, (IQR: 24.17%) between automatic and reference segmentations, which indicates the DL method underestimated the RC with respect to the raters. The median of the absolute volume was 24.7cm^3^ (IQR: 19.1cm^3^) for EE, 26.6cm^3^ (IQR: 26.7cm^3^) for EH, 26.1cm^3^ (IQR: 23.3cm^3^) for MB and 21.7cm^3^ (IQR: 19.6cm^3^) for the automatic segmentation. Figure 2 shows boxplots of DC values, relative volume errors and the absolute volumes for the automatic approach in relation to the experts. According to the Kruskal Wallis test we did not detect a statistically significant difference regarding the distribution of the measured volumes for the different raters and the automatic method (chi-square = 1.46, *p* = 0.69). In contrast, a statistically significant difference in DC (chi-square = 11.63, *p* = 0.04) and relative volume error (chi-square = 22.45, *p* = 0.00043) was found. The result of the subsequent Wilcoxon rank-sum test between rater-to-rater (EE-EH, EE-MB, EH-MB) and automatic-to-rater (Automatic-EE, Automatic-EH, Automatic-MB) pools are shown in Figs. 2 and 3. The automatic segmentation volumes tend to be smaller than the expert volumes, which corresponds with the underestimation found in the relative volume error measurement. The main sources of error by the automatic method were localized to signal inhomogeneity (especially in T2w and FLAIR sequences) and other intensity patterns (edema, subarachnoid space, or ventricles). Figure 4 shows cases representing good and bad performances.

**Table 2:** Comparison of contours

**Pairing**	**DC (IQR)**	**Rel. vol. err. (IQR)**
Automatic-EE	0.83 (0.14)	−0.06 (0.33)
Automatic-EH	0.81 (0.12)	−0.17 (0.29)
Automatic-MB	0.81 (0.13)	−0.09 (0.30)
EE-EH	0.85 (0.08)	−0.11 (0.18)
EE-MB	0.84 (0.07)	−0.08 (0.17)
EH-MB	0.86 (0.07)	0.04 (0.22)

Median and interquartile range of the Dice coefficient (DC) and relative volume errors (Rel. vol. err.) for the three experts (EE, EH, MB) and the automatic approach.

## Discussion

High quality auto-segmentation of the targets and OARs is a very welcome development in RT. Considering the status of innovation of DL methods for auto-segmentation in the brain, post-operative target definition is the key to an implementation of the method for RT purposes. Up to now, some work has been reported for DL-based auto-segmentation of post-operative tumor components of GBM patient [9, 23, 24]. Still missing, was the segmentation of the RC.

With our in-house developed dedicated DL based segmentation method, we have attempted to reproduce the resection cavity as defined by manual contouring of RT experts. The auto-segmented volumes showed a similarity to the reference segmentation with a DC of 0.84. This was only slightly lower than the observed inter-rater variability. The DL method however had a lower robustness and resilience to imaging artefacts such as blood products and air pockets in the resection cavity.

Effective time saving is one of the intended endpoints in the implementation of fully-automated segmentation methods to the RT field. The implementation of such a system for the number of brain tumor patients treated each year and the extension to multiple other treatment sites (e.g. head and neck, lung, prostate, etc.) would result in significant time savings. In total, our proposed method produces an RC segmentation for one case in approximately 90 s, compared to 20.7 (±10.1) minutes for manual contouring. It has to be noted that there will be time required for validation and possible adjustment. Depending on the quality of the segmentations and possible QA system, this time can vary widely. The reduced time could be invested in improving patient care, reducing the treatment costs and increasing the accessibility for patients to high quality radiation therapy. Besides, accurate and efficient auto-segmentation is an important requirement for the innovation of daily adaptive treatments [34].

Automatic segmentation tools that are, or have been incorporated in different treatment planning systems, were mainly atlas based and could not achieve the required level of accuracy. Accordingly, radiation oncologists may spend more time correcting automatic segmentations, than generating manual contours from scratch. This has affected the trust of RT specialist in auto-segmentation and has impeded a more widespread use of auto-segmentation for RT purposes.

The GTV in post-OP glioblastoma patients consists of multiple morphological structures of which the RC is one. In our method, to obtain a target definition these structures are all segmented separately by the DL method. This enables the physician to create and adapt the GTV according to their own preferences and institutional guidelines. In our opinion this would be a more useful and acceptable and yet time saving approach for clinical practice, than to directly have the complete GTV defined by auto-segmentation.

A few other groups have investigated automatic target structure delineation from imaging. Cheng et al. evaluated a level set-based approach for identifying GTV and clinical target volume (CTV) in five glioma patients using post-operative T2w MRI and CT [35]. The reported mean DC was between 0.66 and 0.83, and their results showed a tendency to underestimate the CTV. In our opinion, automated generation of the CTV cannot be compared to auto-segmenting a GTV. The GTV is per definition constructed on medical image morphology, the CTV however, is a medical decision, based on guidelines considering clinical experience and on tumor properties that are not quantifiable on imaging. With the current DL methods based on MRI, CTV definition is not yet feasible. Mazzara et al. assessed fully automated brain MR segmentation methods for RT planning [2]. Both pre- and post-operative images were applied for the target delineation. They reported a larger variation for post-operative cases compared to pre-operatively. They indicated that the margins of residual tumor were unclear on post-operative images, making the identification of the GTV a difficult task for both physicians and auto-segmentation methods. The difficulty in identifying the residual tumor components by physicians is an important issue. Besides Mazzara et al., also Zeng et al. reported on the poor definition of tumor segments in post-operative imaging. From the 88 post-operative scans available in the BRATS database they excluded 56 because of incorrect segmented volumes in the “ground truth” [23]. Recently, Visser et al. reported on the difficulty, even for highly experienced experts, to manually segment GBM on postoperative imaging [8]. This brings up the question of how much of the “ground truth” is actually valid, and therefore in this manuscript we referred to it as the “reference volume”.

**Fig. 5:**
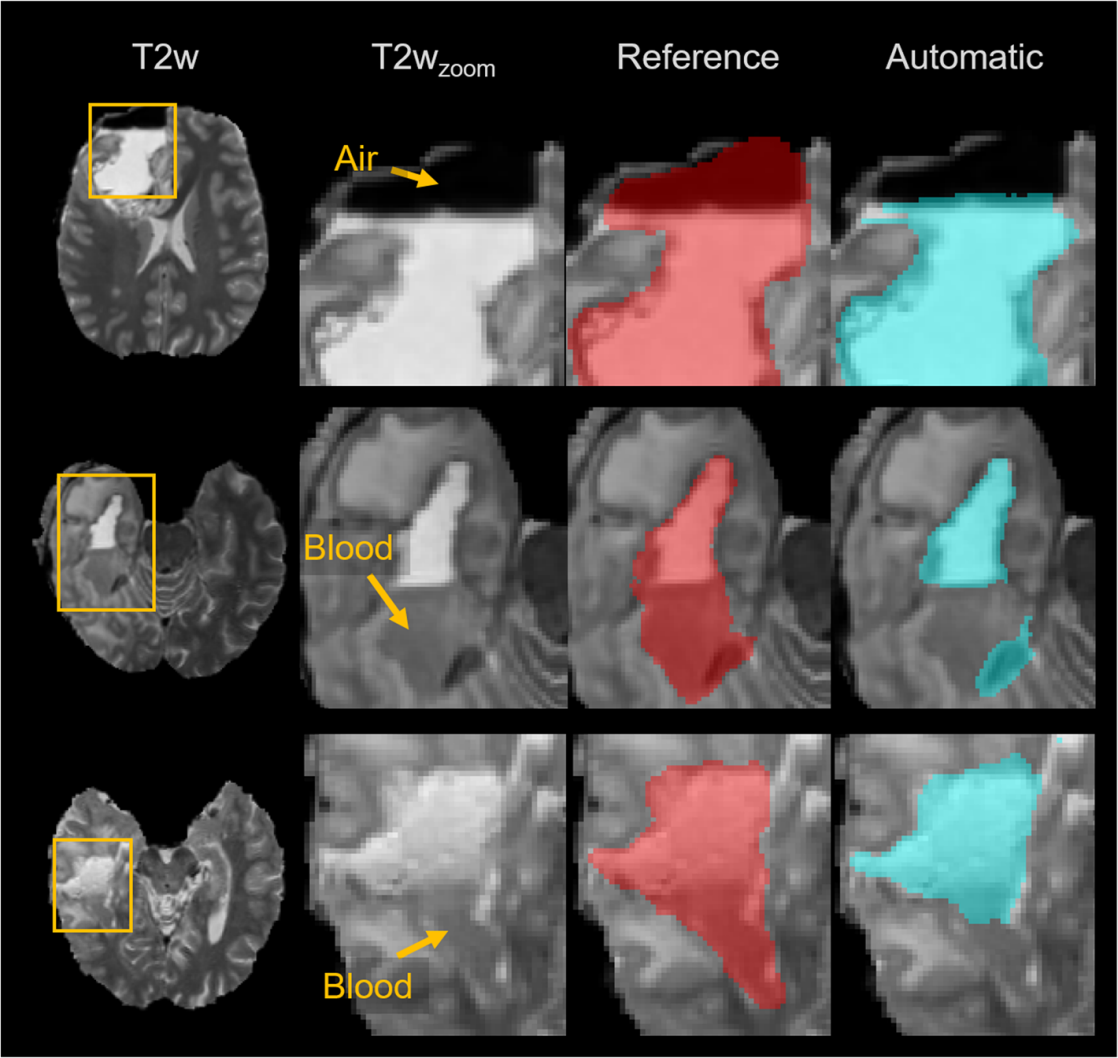
Segmentation errors introduced by air pockets and blood products. The rows indicate erroneous cases and the columns show T2-weighted images, zoomed T2-weighted images, expert consensus segmentation (reference) overlays and automatic segmentation overlays

Although the results are promising, the auto-segmentation is characterized by some typical errors. The observed lower overall volume of the automated approach with respect to the volume of the reference segmentation seems to be mostly caused by specific outlier cases 7, 22 and 23 (Figs. 3 and 4). The divergent results in the outliers are due to a deficiency of the model to identify blood products, air pockets and other deviating MRI intensities, which result in image areas not being included in the RC. Figure 5 illustrates clearly how the automatic segmentation excludes air and blood from the RC. Within the cohort of 30 cases, the occurrence of air pockets and or blood products in the RC were to scarce for proper DL training. A larger cohort and better characterizing these confounding effects, can enable an effective stratification of blood products and air pockets, and lead to improved capability of handling these cases. The small retrospective dataset used for this proof of concept study is also a limitation because it can result in bias in the selected cases. It is a monocentric study and the images are obtained from only two MRI scanners. To obtain a more general validation, a larger prospective multi-institutional dataset is required to confirm the current preliminary results.

Although the CNN architecture might influence the segmentation performance, we expect only subtle differences with other common segmentation architectures, e.g., the U-Net [36]. The employed architecture builds, as most common segmentation architectures, on a base structure consisting of a contraction-expansion path with skip-connections. We used densely connected units within this base structure because it empirically showed faster convergence compared to the U-Net.The evaluation metrics used in our study correspond to the common standard used to evaluate segmentation approaches. However, since the aim is an RT application, it would be valuable to assess the automatic segmentation approach on a more clinically relevant dosimetric level, as has been performed by auto-segmentation studies for the head and neck area [37, 38]. In these studies, they investigated the dosimetric impact on auto-segmented structures versus manual contoured structures in the head and neck area. The difference in the target volume could lead to significant dosimetric differences after RT planning. Conson et al. also reported on the dose-volume effects when using automated segmentations of critical brain structures [39]. Despite there was a volumetric differences between automatically constructed and reference volumes, dosimetric parameters obtained using automated segmentations were comparable with the dosimetry based on the reference contours. In our planned ensuing work, the effect of auto-segmented target and OAR definition on dosimetry will be included as well as incorporating CT imaging.

Many commercial as well as free open source AI based applications are being developed and used in research and proposed for clinical practice. We believe the proof of concept presented in this article, an AI based multimodal MRI solution for tumor cavity segmentation, will contribute to this movement as a unique piece of work. Furthermore, when we move towards an increased usage of said technologies in clinical practice we believe it is important to focus research efforts in the evaluation on the quality of the technology. This includes in particular, novel quality control metrics that are focused towards clinical relevant outcome measures.

This study complements our previous research in pre-, and post-operative brain tumor segmentation [24, 27, 40, 41]. Besides the feasibility of proper auto-segmentation of the OARs and postoperative tumor segments of GBM, we are now able to segment the RC as well, in order to obtain a complete target definition. In this regard, our future work includes developing and integrating a fully automatic segmentation tool for clinical radiotherapy, based on a dedicated DL method.

## Conclusions

We presented a DL approach for automated postoperative RC segmentation. Although the automatic results are subpar to manual contours by RT experts, the results are promising. With the possibility of auto-segmentation of the RC, the radiation target as defined by the international guidelines can now be determined by DL-based auto-segmentation. This last step will pave the way to developing and implementing a fully automated segmentation application for brain RT.

## Data Availability

The imaging data generated and/or analysed during the study are not publicly available due to privacy and confidentiality. Additional data on the DL method or results are available from the corresponding author on reasonable request.

## Abbreviations

3D: Three Dimensional
BRATS: Brain Tumor Image Segmentation Benchmark
CM3: Cubic Centimeter
CNN: Convolutional Neural Networks
CTV: Clinical Target Volume
DC: Dice Coefficient
DL: Deep Learning
EORTC: European Organization for Research and Treatment of Cancer
FLAIR: Fluid Attenuated Inversion Recovery
GBM: Glioblastoma Multiforme
GPU: Graphics Processing Unit
GTV: Gross Tumor Volume
IQR: Interquartile Range
ML: Machine Learning
MRI: Magnetic Resonance Imaging
OAR: Organ at risk
RC: Resection Cavity
RT: Radation Therapy
RTOG: Radiation Therapy Oncology Group
T1w: T1 weighted
T2w: T2 weighted
